# Describing workplace interventions aimed to improve health of staff in hospital settings – a systematic review

**DOI:** 10.1186/s12913-021-07418-9

**Published:** 2022-04-07

**Authors:** Verity Worley, Penny Fraser, Steven Allender, Kristy A. Bolton

**Affiliations:** 1 Global Obesity Centre, Institute for Health Transformation, School of Health and Social Development, Deakin University, Geelong, Australia; 2 Institute for Physical Activity and Nutrition, School of Exercise and Nutrition Sciences, Deakin University, Geelong, Australia

**Keywords:** Workplace intervention, Hospital, Staff, Health behaviours, Systematic review

## Abstract

**Background:**

A large proportion of staff working in hospital settings are overweight or obese, have poor dietary habits and low physical activity levels. The workplace is a priority setting for health promotion. This systematic review will describe dietary and physical activity workplace interventions that have aimed to improve the health of staff in hospital settings; and the barriers and enablers of implementing these interventions.

**Methods:**

A systematic search retrieved 551 studies from 2004 to 2020 using the following databases CINAHL Complete, MEDLINE Complete, Academic Search Complete, Global Health, Health Source Nursing/Academic Edition and PsycINFO. Studies were included if they: (1) took place in a hospital setting; (2) employed a physical activity or dietary intervention to improve the well-being of staff; (3) the intervention duration was 12 weeks or over; (4) used a control group. The Integrated quality Criteria for the Review of Multiple Study designs (ICROMS) and National Institute of Health’s National Heart Lung and Blood Institute Quality Assessment Tools for Observational Cohort and Cross-Sectional Studies tools were used to assess quality of included studies. A narrative review was conducted.

**Results:**

Quality analysis identified six studies of high quality, nine moderate quality, and three low quality. Of these 18 studies, 15 reported at least one positive health outcome. The evidence revealed that multi-component strategies, financial incentives and motivational strategies were the most effective approaches to improve health behaviours of hospital staff.

**Conclusion:**

Hospital-based dietary and physical activity workplace interventions show promise as an effective strategy for improving health behaviours of hospital staff. Methodological limitations highlight the need for more research from high-quality, randomised control trials, to gain further insight into the benefits of workplace interventions in hospital settings.

**Supplementary Information:**

The online version contains supplementary material available at 10.1186/s12913-021-07418-9.

## Background

In 2016, overweight and obesity affected 39% of the adult population worldwide [1] and in high income countries like Australia the overweight and obesity rates climbed to as high as 67% [2]. Obesity is also a well-known risk for chronic type 2 diabetes, cardiovascular disease, high blood pressure, musculoskeletal disorders and some cancers [1]. Despite awareness of modifiable risk behaviours such as physical inactivity, poor nutrition, smoking and alcohol [3], obesity rates persist and consequently aggregate a significant strain on the health care system [4]. Obesity is resistant to prevention and treatment because it is a complex condition driven by a range of factors from individual behaviours to obesogenic built environments [5].

The workplace is one of the settings prioritised for health promotion according to the World Health Organization due to the influence of the workplace on physical, mental, economic and social wellbeing of employees/workers and their families, which extends to the wider community and society [6]. There are benefits of promoting health within the workplace for both the organisation/employer and the employee/worker/staff member (hereon in referred to as staff for simplicity). Given that hospitals are wholly concerned with health, the hospital workplace setting seems a logical site for obesity prevention initiatives for their staff. For hospital staff, the often-stressful nature of the hospital environment which is also characterised by shift work and night work; increases the difficulty to maintain healthy lifestyle behaviours [7, 8].

Research has revealed staff who work in hospital settings have difficulty maintaining a healthy weight, have poor dietary habits, low physical activity levels, high levels of stress and musculoskeletal conditions [9–11]. In the UK, over 40% of healthcare workers were classified as overweight or obese, 45% failed to reach the recommended physical activity guidelines, 30% of employees described their role as sedentary, over 50% of staff were not reaching the daily target of fruit or vegetable servings per day, and over 30% were eating high fat and high sugar foods every day [11]. Furthermore, a multi-country study in Australia, New Zealand and the United Kingdom found that over 60% of midwives were overweight or obese [9].

Key modifiable behaviours to reduce the risk of overweight and obesity are diet and physical activity. Strategies trialled in hospital settings to improve diet have included food labelling in cafeterias [12, 13], healthy catering initiative [14], installing healthier vending machines [13, 15], healthy cookbook, water bottle, sandwich container, educational resources and messaging [16], workplace champions to role model health behaviours [13] and healthy messaging signage [13, 16]. Strategies to increase physical activity have included pedometers [13, 16], education with an online learning tool [17], programs (aerobic, sports) [18], electronic messaging and guided walks [16], and sit-stand desks [19]. To date there is no consensus on the most effective strategies to implement and improve health status of staff in hospital settings, however the strategies above have been promising in improving awareness and knowledge in nutrition [12]; along with improvements in diet [14–16] and physical activity behaviours [13, 16, 18, 19]. A recent review has revealed organisational barriers to healthy eating in nurses such as long work hours, shift work, high workload, low staffing and work breaks being too short or too few [20]. Environmental barriers (e.g. access/cost of unhealthy food, inadequate food storage and preparation facilities), social barriers (e.g. access to treat food, social unhealthy eating practices, stress-related eating) and individual behaviours (e.g. lack of knowledge, self-efficacy and motivation) were also reported [20].

Despite the clear need to improve the health of hospital staff, i.e. dietary consumption of fruit and vegetables and increase physical activity levels; there is no clear consensus on which intervention strategies produce the best outcomes, or recognition of the barriers and enablers for implementing these dietary and physical activity workplace interventions in hospital settings [21]. There has been some review and synthesis of trials targeting stress levels of nurses and health professionals [21–23], burnout in emergency nurses and mental health professionals [24, 25], and the ability to increase physical activity in other settings [26]. One systematic review examined the promotion of diet and physical activity in nurses, and whilst highlighting the lack of interventions to promote diet and physical activity in nurses; its inclusion criteria applied to a wider health care setting (e.g. hospital, academic medical centres, university, surgical units, long-term facilities), included all durations of interventions, included studies without control groups and did not examine barriers and enablers related to the included studies [21].

There are no systematic reviews that examine improving health behaviours of staff through diet and physical activity purely in a hospital setting, or that extended beyond nurses to all health staff; nor understanding about the barriers and facilitators of implementing these types of interventions in a hospital setting. Given the diverse range of occupations within the hospital setting, it is important we extend our health promotion focus beyond nurses alone. Furthermore, other reviews highlight the lack of quality studies found in their reviews [21, 27], which shaped this review to target higher quality evidence, for example interventions with control groups and intervention durations considered adequate to influence behaviour change. This systematic review therefore describes workplace interventions that have aimed to improve health of staff in hospital settings and the barriers and enablers that influence the success of those interventions. Understanding the breadth of workplace interventions is needed to set the next platform of initiatives towards preventing obesity.

## Aims and objectives

Research questions to be addressed in this systematic review:

1. What dietary and physical activity interventions have aimed to improve diet, physical activity in staff, specifically in a hospital setting, compared to no intervention?
2. What are the barriers and enablers of implementing these types of workplace interventions in a hospital setting?

## Methods

### Registration

This systematic review was conducted respecting the Preferred Reporting Items for Systematic Reviews and Meta-Analyses (PRISMA) protocols [28]. The protocol for this review was registered and published in the International Prospective Register of Systematic Reviews on 15 August 2018, PROSPERO registration CRD42018096797.

### Data sources and search strategy

The search terms were agreed upon through consultation between the co-authors and Deakin University’s research librarian who had expertise in systematic reviews. The terms were categorised against a methodological approach, using the PICO (patient or problem; intervention, control or comparison, outcome) method as reference [29].

The final search terms are as follows: nutrition* or diet* or “physical activ*” or exercis* AND (workplace or worksite) and (health* or wellness or program* or intervention of “health promotion”) AND staff or employee* or provider* or nurse* or doctor* or midwi* or “shift work” AND Survey* or questionnaire* or “randomi?ed control trial” or stud* or interview* or “focus group*” AND “primary health*” or hospital* NOT patient*.

Note: * at the end of keywords will find variations of the word;? *will look for alternate spelling of the word* [30]*.* Refer to Supplementary Table 1 for search strategy outline.

Systematic searching of the literature was conducted across electronic databases CINAHL Complete, MEDLINE Complete, Academic Search Complete, Global Health, Health Source Nursing/Academic Edition and PsycINFO, between 3 April 2018 and 17 June 2020. Only peer reviewed, published articles, in English, from 1st January 2004 to 17 June 2020 and generated 551 results.

### Inclusion and exclusion criteria

Only peer reviewed, published articles, from 2004 onwards were included in the review. This time frame coincides with the World Health Organization’s *Global Strategy on Diet, Physical Activity and Health* and when interventions started to become more prevalent in the workplace [31]. Eligible studies met the following criteria: (1) included participants who worked in hospital settings; (2) implemented dietary or physical activity intervention in the workplace; (3) reported on diet (fruit, vegetable, high fat and sugary foods), physical activity, anthropometric measurements, blood pressure, stress levels, or musculoskeletal outcomes. Stress and musculoskeletal outcomes were included given the potential relationship with stress, shift work and unhealthy eating practices in nurses [32] and the link between physical activity and musculoskeletal outcomes [33]. All studies were included except for reviews.

Studies were excluded if; staff and patients were combined as one intervention group and were not able to be disaggregated into the staff subpopulation only; the study involved multiple worksites including a hospital but failed to distinguish between the worksite settings in their analysis and reporting of findings; the setting was outside of a hospital; the outcomes focused on perceptions of an intervention; interventions took place in community settings; outcomes were not included in the inclusion criteria; there was no control group reported; the intervention was under 12 weeks duration as 12 weeks is suggested as a minimum length to see behaviour changes from an obesity prevention intervention [34]; did not report primary data; the publication was a review and if the study was not reported in English.

### Study selection

A systematic review software, Covidence [35], was used to screen the electronic articles and keep record of the results. Two authors, VW and KAB, separately conducted screening of the titles and abstracts against the inclusion/exclusion criteria. All studies included for full text screening had their references scanned by VW and nine additional studies were located for screening. Any conflicts that arose from the screening process were reviewed by both authors again until all conflicts could be resolved. Included studies underwent a full text screening, independently by VW and KAB, to further ascertain that the chosen studies met the inclusion criteria.

### Data extraction and quality assessment of studies

Included studies had their key characteristics extracted and were collated into Table 1. Data was extracted by one reviewer (VW) and checked for accuracy and completeness by a second reviewer (KAB). The Integrated quality Criteria for the Review Of Multiple Study designs (ICROMS) tool was used for the quality assessment (Table 2) in this review as it covers multiple study designs (randomised control trials, non-randomised control trials, controlled before-and-after, non-controlled before-and-after studies), yet it draws on thorough criteria for its analysis [36]. For the cross-sectional study that fell outside of the ICROMS tool, the National Heart and Blood Institute Quality Assessment Tool for Observational Cohort and Cross-Sectional Studies was used [37]. VW and KAB conducted the quality analysis independently. Any disagreements were resolved by discussion until a consensus decision was made.

**Table 1:** Summary of studies meeting the inclusion criteria

Study	Sample size	Country	Target population	Intervention components	Intervention length	Follow-up duration	Outcome measures	Findings (Between-group differences)
**Randomised Control Trial**								
Barene [18] 2014	118	Norway	Female nurses and nursing assistants, however participation offered to other hospital employees	IG: Group 1, soccer; Group 2, zumba	40 weeks	Baseline	VO2 max	– Soccer, + Zumba
						40 weeks	Blood pressure	–
				CG: No intervention.			Weight BMI Body fat	– Soccer+ Zumba – Soccer+ Zumba +
Hewitt [38] 2008	20	United Kingdom	Staff from cytology laboratory	IG: Progressive aerobic exercise program, 4 times a week for 8 weeks and to continue without further advice for another 4 weeks.	12 weeks	Baseline	Blood pressure	–
						4 weeks	BMI	–
						8 weeks	VO2max at 2 min	+
				CG: Maintained their current physical activity level.		12 weeks	VO2max at 4 min	+
							Biological markers (fasting glucose, total cholesterol)	–
Kullgren [39] 2013	105	United States of America	Hospital employees	IG: 24 weeks of monthly weigh ins. Group 1, individual financial incentive of $100 per month; Group 2, group financial incentive of $500 per month (split between group members who hit their target).	36 weeks	Baseline	PA in last seven days	–
						4 weeks	Restraint in eating	+
						8 weeks	Weight loss BMI	+ +
						12 weeks	Other weight loss mediators	–
						16 weeks		
						20 weeks		
						36 weeks		
				CG: Website access.				
Lemon [40] 2010	806	United States of America	Employees working at least 20 h/week	IG: Social marketing campaign (newsletter, website, educational materials), StairWELL campaign, physical activity challenges, nutritional signage, healthy menu options.	2 years	Baseline 12 months 24 months	BMI	–
							Perceptions of organisational commitment to employee health	+
							Perception of employee normative behaviours	–
				CG: No intervention				
Low [41] 2015	57	United States of America	Female health care workers	IG: Weekly communication on goal setting and, overcoming goals.	6 months	Baseline 6 months 1 year	Days per week of exercise	+/−
							Readiness to change	–
							Triglycerides	+
				CG: Offered classes on (weight/diet, stress, exercise and smoking cessation), gym access and organised walks			Exercise intensity	+/−
							Weight loss	+/−
							Stress	+/−
Lowe [42] 2010	96	United States of America	Hospital employees	IG: A pricing incentive to purchase low-energy-density foods and education about nutrition in 4 information sessions.	3 months	Baseline 1 month 2 months 3 months 6 months 12 months	Energy intake	–
							Nutrient intake	–
							Food selection	–
				CG: 10 new low-energy-density foods and labelling of food at lunch.			Food intake outside of cafeteria	–
							Weight BMI	– –
							Cholesterol	–
Matsugaki [43] 2017	30	Japan	Female nurses	IG: Supervised aerobic and resistance training (24 sessions total).	12 weeks	Baseline	VO2max	+
						12 weeks	Muscle strength	+ 180 deg/sec knee extensions
				CG: Voluntary exercise.			Mental health	–
							BMI	–
							Biochemical data	+ reactive oxygen metabolites
Ostbye [44] 2015	550	United States of America	Employees with obesity	IG: Extensive behavioural coaching.	14 months	Baseline	Weight BMI	– –
						14 months	Fruit and vegetable servings	+/−
				CG: Educational intervention.			% energy from fat	+
							MVPA	–
							Sedentary time	–
							Exercise and nutrition goals and planning	–
							Sugar sweetened beverages intake	–
Palumbo [45] 2012	11	United States of America	Nurses > 45 years old	IG: Tai Chi classes once a week, 10 min practice unsupervised for at least 4 days.	15 weeks	Baseline	Workplace injury	+/−
						15 weeks	Time off	+/−
							Musculoskeletal injury	–
							Physical and mental well-being	+ functional reach
				CG: No classes.			Work productivity	+/−
Stenner [46] 2020	265	Germany	Middle-aged sedentary women working at a hospital	IG: Endurance training exercise intervention	6 month	Baseline 6 months	Cardiorespiratory fitness (VO_2 peak_) Body weight Body fat % BMI Work Ability Index total score PA total score PA sports score	+ + + – + (item 2 only) + +
				CG: No intervention				
Strijk [47] 2012	730	Netherlands	Hospital employees working at least 16 h/week	IG: A vitality exercise program, provision of free fruit and three visits to a Personal Vitality Coach.	6 months	Baseline 6 months	PA	+
							MVPA	–
							Fruit intake	+
				CG: Written information about a healthy lifestyle			VO2max	–
							Mental health	–
							Need for recovery	+
Thorndike [48] 2012	330	United States of America	Hospital employees	IG: Internet website about goal setting, self-monitoring and behavioural support	1 year	Baseline	Weight loss BMI	+/− +/−
						10 weeks	Fruit and vegetable servings	+/−
						12 months	Fatty food intake	+/−
				CG: No intervention			Sugary food intake	+/−
							PA	+/−
**Controlled Before-After**								
Borg [49] 2010	332	Australia	Inactive health service employees	IG: CG intervention plus four additional newsletters	12 months	Baseline	Walking minutes per week,	–
						12 months	MVPA minutes per week	+
				CG: ‘Step by Step’ guidebook, diary cards, pedometer, brochure.			PA per week	–
							Proportion meeting public health recommendations	–
Hamm [50] 2019	38	Canada	Hospital employees	IG: Physical activity counselling plus tailored exercise prescription	4 months	Baseline 2 months 4 months	BMI Accelerometry Overall fitness score Grip strength Push-ups Leg power Balance Trunk endurance Sit and reach	– – + – + – – – +
				CG: Physical activity counselling				
LaCaille [13] 2016	500	United States of America	Hospital employees	IG: Pedometer distribution, StairWELL campaign, labelling all foods in worksite cafeteria and vending machines, traffic light system, influential employees, persuasive messaging throughout the hospital.	12 months	Baseline 6 months	BMI Weight loss	– –
						12 months	Waist circumference	–
							Physical activity	+
							Fruit and vegetable servings	+
							Knowledge about energy balance	–
							Perceptions of employer’s commitment to well-being	+
				CG: No intervention				
Sallon [51] 2017	215	Israel	Hospital employees	IG: CCG intervention (Cognitive, somatic, emotive-expressive, dynamic-interactive and hands-on). 75 h of instruction in 30 weekly sessions and 1-day workshop.	8 months	Baseline	Personal accomplishment	–
						8 months	Perceived stress	–
							Burnout	+
				CG: No intervention			Job-related tension	+
							Work productivity	+
							General health and mood	+
Yuan [52] 2009	90	Taiwan	Nurses	IG: Exercised on stair-stepper for 20-30 min per day (70–85% max heart rate), at least three times a week.	3 months	Baseline	BMI	+
						3 months	Grip strength	+
							Flexibility	+
							Abdominal muscle durability	+
				CG: Performed usual work habits.			Back muscle durability	+
							Cardiopulmonary durability	+
							Blood pressure	–
**Cross-sectional**								
Geaney [14] 2011	100	Ireland	Hospital employees consuming at least one meal in the hospital staff canteen daily	IG: Catering intervention, salt removed from tables, nutritional information display in cafeteria and encouraged to eat healthy foods.	2 years	Over the 2 years	Energy intake	+
							Fat intake	+
							Salt intake	+
							Sugar intake	+
				CG: No intervention.				

Notes: *IG* intervention group; *CG* control group; *PA* physical activity; *BMI* body mass index; +, a positive significant outcome; (i.e. intervention was significantly effective on the outcome); +/−, non-significant marginal change or no *p*-value reported; −, no significant effect on the outcome (i.e. intervention was not effective on the outcome)

**Table 2:** ICROMS [36] quality analysis on studies meeting the inclusion criteria

	Barene [18]	Hewitt [38]	Kullgren [39]	Lemon [40]	Low [41]	Lowe [42]	Matsugaki [43]	Ostbye [44]	Palumbo [45]	Stenner [46]	Strijk [47]	Thorndike [48]	Borg [49]	Hamm [50]	LaCaille [13]	Sallon [51]	Yuan [52]
	2014	2008	2013	2010	2015	2010	2017	2015	2012	2020	2012	2012	2010	2019	2016	2017	2009
**1. Clear aims and justification**																	
A. Clear statement of the aims of the research	**✓✓**	**✓✓**	**✓✓**	**✓✓**	**✓✓**	**✓✓**	**✓✓**	**✓✓**	**✓✓**	**✓✓**	**✓✓**	**✓✓**	**✓✓**	**✓✓**	**✓✓**	**✓✓**	**✓✓**
**2. Managing bias in sampling or between groups**																	
A. Sequence Generation	**✓✓**	**✓✓**	**✓✓**	**✓**	**✓**	**✓✓**	**✓✓**	**✓✓**	**✓**	**✓✓**	**✓✓**	**✓✓**	N/A	N/A	N/A	N/A	N/A
B. Allocation Concealment	**✓✓**	**✓✓**	**✓✓**	**✓**	**✓**	**✓**	**✓✓**	**✓**	**✓**	**✓✓**	**✓✓**	**✓**	N/A	N/A	N/A	N/A	N/A
D. Intervention and control group selection designed to protect against systematic difference/selection bias	N/A	N/A	N/A	N/A	N/A	N/A	N/A	N/A	N/A	N/A	N/A	N/A	**✓**	**✓✓**	**✓✓**	**✗**	**✗**
**3. Managing bias in outcome measurements and blinding**																	
A. Blinding	**✓**	**✗**	**✓✓**	**✓**	**✓**	**✗**	**✓✓**	**✓**	**✓**	**✓✓**	**✓✓**	**✓**	N/A	N/A	N/A	N/A	N/A
B. Protection against selection bias	N/A	N/A	N/A	N/A	N/A	N/A	N/A	N/A	N/A	N/A	N/A	N/A	**✓✓**	**✓✓**	**✓✓**	**✓✓**	**✓✓**
C. Protection against contamination	N/A	N/A	N/A	N/A	N/A	N/A	N/A	N/A	N/A	N/A	N/A	N/A	**✓**	**✓✓**	**✓✓**	**✓**	**✓**
E. Protection against detection bias	**✓✓**	**✓✓**	**✓✓**	**✓✓**	**✓✓**	**✓✓**	**✓✓**	**✓✓**	**✓✓**	**✓✓**	**✓✓**	**✓✓**	**✓✓**	**✓✓**	**✓✓**	**✓**	**✓✓**
F. Reliable primary outcome measures	**✓✓**	**✓✓**	**✓**	**✓✓**	**✓✓**	**✓✓**	**✓✓**	**✓✓**	**✓✓**	**✓✓**	**✓✓**	**✓✓**	**✓✓**	**✓✓**	**✓✓**	**✓✓**	**✓✓**
**4. Managing bias in follow-up**																	
A. Follow-up of subjects (protection against exclusion bias)	**✗**	**✓**	**✓✓**	**✓✓**	**✓**	**✗**	**✓✓**	**✓✓**	**✗**	**✓✓**	**✗**	**✗**	N/A	N/A	N/A	N/A	N/A
B. Follow-up of patients or episodes of care	**✗**	**✓✓**	**✓✓**	**✓✓**	**✓**	**✗**	**✓✓**	**✓✓**	**✗**	**✓✓**	**✓**	**✓✓**	N/A	N/A	N/A	N/A	N/A
C. Incomplete outcome data addressed	**✓✓**	**✓**	**✓✓**	**✓**	**✓✓**	**✓**	**✓**	**✓✓**	**✓**	**✓✓**	**✓✓**	**✓✓**	**✓✓**	**✓✓**	**✓✓**	**✓**	**✓**
**5. Managing bias in other study aspects**																	
A. Protection against detection bias: Intervention unlikely to affect data collection	**✓**	**✓**	**✓**	**✓**	**✓**	**✓**	**✓**	**✓**	**✓**	**✓**	**✓**	**✓**	**✓**	**✓**	**✓**	**✓**	**✓**
**6. Analytical rigour**																	
C. Analysis sufficiently rigorous/free from bias	**✓✓**	**✓✓**	**✓✓**	**✓✓**	**✓✓**	**✓✓**	**✓✓**	**✓✓**	**✓**	**✓✓**	**✓✓**	**✓✓**	**✓✓**	**✓✓**	**✓✓**	**✓✓**	**✓✓**
**7. Managing bias in reporting/ethical considerations**																	
A. Free of selective outcome reporting	**✓✓**	**✓✓**	**✓✓**	**✓✓**	**✓✓**	**✓✓**	**✓✓**	**✓✓**	**✓✓**	**✓✓**	**✓✓**	**✓✓**	**✓✓**	**✓✓**	**✓✓**	**✓✓**	**✓✓**
B. Limitations addressed	**✓✓**	**✓**	**✓✓**	**✓✓**	**✓✓**	**✓**	**✓✓**	**✓✓**	**✓✓**	**✓✓**	**✓✓**	**✓**	**✓✓**	**✓✓**	**✓✓**	**✓**	**✓✓**
C. Conclusions clear and justified	**✓✓**	**✓✓**	**✓✓**	**✓✓**	**✓✓**	**✓**	**✓✓**	**✓✓**	**✓✓**	**✓✓**	**✓✓**	**✓✓**	**✓✓**	**✓✓**	**✓✓**	**✓✓**	**✓✓**
D. Free of other bias	**✓**	**✓✓**	**✓✓**	**✓**	**✓**	**✗**	**✓✓**	**✓**	**✗**	**✓✓**	**✓✓**	**✓✓**	**✓✓**	**✓✓**	**✓✓**	**✓**	**✓**
E. Ethics issues addressed	**✓✓**	**✓✓**	**✓✓**	**✓✓**	**✓✓**	**✓✓**	**✓✓**	**✓✓**	**✗**	**✓✓**	**✓✓**	**✓✓**	**✓✓**	**✓✓**	**✓✓**	**✓✓**	**✓✓**
**Met mandatory criteria**																	
	✗	✗	**✓**	✗	✗	✗	**✓**	✗	✗	**✓**	**✓**	✗	✗	**✓**	**✓**	✗	✗
**Met minimum score (≥22 if RCT, ≥18 if CBA)**																	
Score	25	26	30*	26	25	19	30*	28	18	30*	28*	26	25	27*	27*	20	22

Legend ✓✓ criterion met; ✓ unclear whether criterion met; ✗ criterion not met; N/A: not applicable to the study design in question

*RCT* randomised controlled trial; *CBA* controlled before after; * meets both mandatory criteria and minimum score

### Data synthesis and analysis

Firstly, the table of key characteristics was used to assess any commonalities and differences between the studies. Secondly, the quality analysis was used to compare the strengths and weaknesses between the studies. Thirdly, a narrative synthesis was conducted, assessing the interventions by strategy.

## Results

### Search and selection findings

**Fig. 1:**
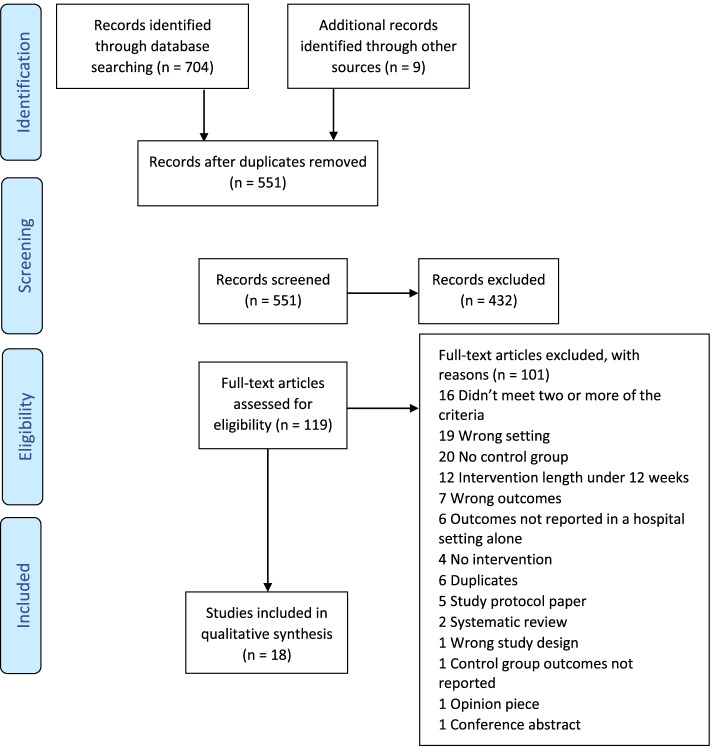
Screening the search results against the inclusion/exclusion criteria

The search generated 551 journal articles. Following the title and abstract screening, the full text of 119 articles were screened against the inclusion/exclusion criteria. This resulted in 18 articles progressing to quality assessment (see Fig. 1) for inclusion in the review corpus.

### Summary of studies

Studies included in the review consisted of five controlled-before-after studies (CBA), 12 randomised control trials (RCT) and one cross-sectional study (Table 1). Eight studies were from United States of America along with one study from each of Canada, Germany, Norway, United Kingdom, Japan, Netherlands, Australia, Israel, Taiwan and Ireland. The sample sizes ranged from 11 to 806 participants. The lengths of the interventions ranged from 3 months to 24 months and targeted combinations of interventions aiming to improve nutrition, physical activity, weight control, musculoskeletal injury risk and stress among hospital staff.

### Quality assessment findings

Questions score two marks for meeting the quality criteria, one mark if the information is unclear, and 0 if the criteria has not been met. The ICROMS decision matrix was used to identify if studies had met the minimum criteria, the mandatory criteria, as well as a global score, to determine if the study was of high quality (Table 3) [36]. Six studies were considered of high quality, and met the ICROMS mandatory and global criteria for their respective study designs [13, 39, 43, 46, 47, 50]. Nine studies met the minimum score but failed to meet the mandatory criteria [18, 38, 40, 41, 44, 48, 49, 51, 52] and two studies were low quality [42, 45].

**Table 3:** Quality analysis [37] of the cross-sectional study meeting the inclusion criteria

	Geaney 2011 [14]
**Question**	**Criteria**
1. Was the research question or objective in this paper clearly stated?	✓
2. Was the study population clearly specified and defined?	✓
3. Was the participation rate of eligible persons at least 50%?	?
4. Were all the subjects selected or recruited from the same or similar populations (including the same time period)? Were inclusion and exclusion criteria for being in the study prespecified and applied uniformly to all participants?	✓
5. Was a sample size justification, power description, or variance and effect estimates provided?	✗
6. For the analyses in this paper, were the exposure(s) of interest measured prior to the outcome(s) being measured?	✗
7. Was the timeframe sufficient so that one could reasonably expect to see an association between exposure and outcome if it existed?	✗
8. For exposures that can vary in amount or level, did the study examine different levels of the exposure as related to the outcome (e.g., categories of exposure, or exposure measured as continuous variable)?	N/A
9. Were the exposure measures (independent variables) clearly defined, valid, reliable, and implemented consistently across all study participants?	✗
10. Was the exposure(s) assessed more than once over time?	✗
11. Were the outcome measures (dependent variables) clearly defined, valid, reliable, and implemented consistently across all study participants?	✗
12. Were the outcome assessors blinded to the exposure status of participants?	✗
13. Was loss to follow-up after baseline 20% or less?	N/A
14. Were key potential confounding variables measured and adjusted statistically for their impact on the relationship between exposure(s) and outcome(s)?	✗
**Overall quality rating**	**Poor**

✓ yes; ✗ no;? couldn’t determine; *N/A* not applicable

The quality analysis for the cross-sectional study by Geaney et al. [14] was analysed with the National Institutes of Health’s National Heart Lung and Blood Institute Quality Assessment Tool for Observational Cohort and Cross-Sectional Studies [37], was also low quality (Table 3). Questions 1, 2, and 4, regarding clear aims, study population, selection process, time period, and prespecified inclusion/exclusion criteria, were strong. There was no sample size justification mentioned and no baseline measures were conducted. Time frame, repeated exposure assessment, outcome measures, blinding of outcome assessors and statistical analyses were all insufficient and data was completed by self-report. Question 8 and 13, regarding exposure levels and loss to follow-up, were not applicable. Question 3 on participation rate could not be determined. The overall rating for this study was poor.

The ICROMS tool revealed that managing bias in sampling or between groups proved challenging for the RCT studies with nine producing adequate sequence generation [18, 38, 39, 42–44, 46–48] and six studies implementing adequate allocation concealment [18, 38, 39, 43, 46, 47]. Four RCTs used blinding to manage bias in their outcome measures [39, 43, 46, 47], five RCTs completed reliable follow-up of participants to protect against exclusion bias [39, 40, 43, 44, 46] and five studies did not address this issue [18, 42, 45, 47, 48]. All of the CBA studies used baseline measures to protect against selection bias however only two CBA studies employed a method to protect against contamination between study groups [13, 50].

Process analysis was adequately presented in 16 of the 17 RCT/CBA studies. Characterised as: sufficiently rigorous, implemented reliable outcome measures, provided clear and justified conclusions, blinded assessment of their primary outcome measures, and addressed ethical issues. All the studies addressed the issue of incomplete data and were free from selective outcome reporting. Although none of the studies explicitly reported that they protected against detection bias, none of the interventions were likely to affect data collection. Thirteen studies addressed their limitations adequately [13, 18, 39–41, 43–47, 49, 50, 52], nine studies were free from other forms of bias [13, 38, 39, 43, 46–50], and two studies had a high risk of other bias [42, 45].

### Diet-related interventions

Two studies focused on interventions to decrease the intake of unhealthy food and drinks from their hospital cafeteria [14, 42]. A RCT by Lowe et al. [42] used food labelling, subsidies on low-energy-density foods, addition of more healthy foods to the menu, and four nutritional educational sessions to increase the intake of low-energy-dense foods. Their results showed no significant between-group differences (the difference between the control group and intervention group) but reported significant decrease in energy intake, fat intake and increase in energy from carbohydrates, across both groups. It is possible that the between-group differences were minimised as both groups received a baseline intervention, a coloured food labelling system paired with an increased selection of healthy food items such as steamed vegetables or low-fat options. Geaney et al. [14] conducted a repeat cross-sectional study of a catering initiative that saw significant decreases in consumption of fat, salt and sugar in the intervention group compared with the control group. These findings coincide with those by Gorton et al. [15] who found their environmental strategy, installing healthier vending machines in hospitals, produced decreases energy, fat and sugar intake.

### Physical activity interventions

Six studies tested physical activity promoting strategies including supervised/unsupervised fitness classes [18], aerobic exercise training [38, 52], endurance training [46], resistance training classes [43], Thai Chi [45], pedometers [13, 49], personalised physical activity counselling [50] and educational materials [49]. Four RCTs showed significant improvements in oxygen uptake performance compared to controls through aerobic exercise classes, resistance training and endurance training [18, 38, 43, 46].

Twelve studies measured body mass index (BMI), of which three found significant decreases in BMI [18, 39, 52]. None of the studies reported significant differences in blood pressure. Matsugaki et al. [43] and Stenner et al. [46] met all the mandatory criteria from the quality assessment however Matsugaki’s study had a small sample size and the findings regarding improved fitness levels in the supervised exercise group may not be generalisable. Stenner et al. [46] revealed some positive improvements in fitness and work ability index in the intervention (endurance training) group, however most of the participants in the study were sedentary and there was a high drop out in the intervention compared to the control group. Hewitt et al. [38] and Yuan et al. [52] also had small sample sizes. Borg et al. [49] showed no between-group differences of walking minutes completed and total physical activity however did find a significant increase in moderate-to-vigorous physical activity (MVPA) in the intervention group compared to the control group, as well as a positive association with pedometer use and meeting public health recommendations [14]. Palumbo et al. [45] found that Tai Chi improved all outcome measures across the board; no time-off taken at work, an increase in productivity, and improvements in functional reach; however not at a significant level. Hamm et al. [50] showed that a personal physical activity counselling program may improve physical activity over 4 months, but it was dependent on those individuals with gym memberships.

### Diet-related and physical activity interventions

Six studies focused on interventions consisting of both nutrition and physical activity outcome measures and all had large participant numbers [13, 39, 40, 44, 47, 48]. Five of these studies took place in USA [13, 39, 40, 44, 48]. Interventions included social marketing campaigns, the StairWELL campaign, physical activity challenges, motivational coaches, educational materials and seminars, website access, pedometers, food labelling and nutritional signage [13, 39, 40, 44, 47, 48]. LaCaille et al. [13], Kullgren et al. [39] and Strijk et al. [47] were high quality studies and the remaining three studies were of moderate quality.

LaCaille et al. [13] showed that their pedometers and StairWELL campaign, combined with food labelling, failed to produce significant between-group differences in BMI, however did significantly increase knowledge on dietary and physical activity behaviours. Lemon et al. [40] also found that their social marketing and StairWELL campaign failed to produce significant impact on BMI. Strijk et al. [47] observed a positive significant difference in fruit intake, need for recovery and sports activity, but not in MVPA, VO2max, or mental health, from their intervention. Kullgren et al. [39] included a financial incentive component which produced a significant decrease in weight compared to baseline in one intervention group but showed no significant differences in dietary or physical behaviours. Østbye et al. [44] found marginal changes in diet and physical activity and Thorndike et al. [48] found a decrease in body weight and improvements in dietary and physical activity behaviours, although no significant between-group differences.

### Diet and/or physical activity and stress reduction interventions

Two studies implemented interventions consisting of gym access, workshops (on weight, diet, physical activity, stress), goal setting and motivational counselling [41, 51]. Low et al. [41] saw minimal changes in stress levels, exercise intensity and weight loss compared to the control group, from their intervention of goal setting and motivational counselling. Sallon et al. [51], a study from Israel, found that their intervention group who attended weekly sessions on topics such as mindfulness, relaxation, body awareness, listening circles, and trigger point massage, displayed a significant increase in their physical well-being, mental well-being, energy, ability to relax, ability to cope with stress inside and outside of the workplace.

### Barriers and enablers

Analysis of the included studies revealed enablers and barriers to improving health behaviours of hospital staff. Enablers included physical activity programs being run outside of work hours [18]; during work hours [46]; the use of workplace health club [46]; supervision influencing higher adherence to training sessions [38] and intensity (but not adherence) [43]; financial incentives [39]; regular (e.g. weekly) encouragement [41]; regular communication [49]; tailored programs [46, 47]; supervised exercise programs [38, 43, 46]; motivational/counselling [41, 50, 51]; coaching [44, 47, 49]; influential employees [13]; persuasive messaging [13, 40]; pedometers [13]; convenience of accessing a program based on the internet [48].

Lack of time to participate was a common barrier [40, 43]. Other reported barriers included stress, limited flexibility during to participate (e.g. particularly for clinical staff/patient centred care staff), staffing challenges, shift work (i.e. shift workers potentially isolated from participating), organisational culture and environment [40], long commute times to and from work [41], initial high motivation to participate but in reality found it difficult to comply/continue with the program [41], environmental changes were slow [13], cost to employers to make changes [13], and some employers feared suggested changes may upset employees/customers [13]. High intensity activities resulted in a decrease in participation and were associated with more participant complaints about heavy workload [40, 43].

## Discussion

### Main findings

This systematic review sought to describe workplace interventions aiming to improve the health of staff in hospital settings. There were 18 studies that met the inclusion criteria in the study period between 2004 and 2020. Several studies were able to generate changes in modifiable risk among hospital staff for knowledge on dietary and physical activity behaviours, diet (fruit intake), physical activity (including cardiorespiratory fitness, fitness scores, flexibility), anthropometry (weight, body fat). In contrast, several studies were unable to produce significant improvements diet (energy intake, fat intake), physical activity (e.g. walking minutes, MVPA, VO2max, exercise intensity), anthropometry (weight), mental health, and stress. Reducing BMI proved difficult with the majority of studies unable to produce a significant decrease in BMI; and none of the studies reported significant differences in blood pressure. Six high quality studies [13, 39, 43, 46, 47, 50] provided strong evidence that workplace interventions in a hospital setting can increase in knowledge about health behaviours, improve diet, physical activity, fitness and cardiorespiratory fitness, and decrease body weight and body fat. The review uncovered numerous strategies employed to increase positive health behaviours in hospital settings focusing on diet and physical activity in isolation; or combined. The strategies included physical activity-based, financial, environmental, motivational, educational or multi-component. Intervention and study design quality were imperative to both the success and the evaluation of these interventions. Previous evaluations have suggested that low quality studies often produce inconsistent findings [21, 27] or may underestimate the intervention effects [53], compared to reviews with moderate to high quality studies that often achieve significant outcomes [54].

### Reported enablers in the studies examined

#### Motivational strategies and counselling

Strategies that were affective in motivating change included role modelling, persuasive messaging, counselling, coaches and supervised exercise programs. Influential employees improved staff health behaviours and produced a flow on effect that continued after the intervention, for example requesting policy changes to install bike racks [13]. Hamm et al. [50] reflected that participant choice into the group activity facilitated better adherence and reflects life in the real world; and the ability to tailor counselling based upon Transtheoretic Model of Behaviour Change to be key enablers. A meta-analysis of workplace interventions in other settings agreed that motivational strategies increased physical activity [55]. Similarly, in this review there was good evidence that the inclusion of a motivation strategy could enhance positive outcomes. Personal, tailored guidance, with regular contact was thought to be a strong influence on compliance and motivation as problems could be detected and solved early [46]. This is similar to other research in primary care settings which have demonstrated motivational strategies to increase physical activity levels [56] and hospitals utilising motivational strategies being more likely to implement policies and practices more successfully [57].

#### Financial reward

Positive health behaviours can be increased through financial incentives, via subsidies of healthy food and drink options, or through financial reward for performing healthy behaviours such as exercise [39, 58]. Group incentives, where a financial reward was shared between group members, were more effective than individual incentives (no sharing of the reward), at improving healthy behaviours [39]. This finding was unique in that other studies have focused on individual rewards and subsidies [54, 59]. Financial incentive studies are scarce in the literature but are often largely effective, with subsidies of 35 to 50% shown to produce significant increases in fruit and vegetable purchases [54, 58]. This review found that a subsidy was unable to produce significant improvements in fruit and vegetable consumption, however the included subsidy intervention was implemented on top of an already established intervention (food labelling and introduction of low-energy-dense food options). This result may have caused “background noise”, dampening down their between-group intervention effects [60]. Furthermore, Finkelstein et al. [61] caution that although financial incentives appear to be effective, they often fail to maintain healthy behaviours once the incentives have ceased. However, financial interventions have been successful in other health areas such as smoking cessation [59]. To ensure long term effectiveness of workplace interventions, sustainability of positive behaviour change would also need to be addressed.

#### Multi-component interventions

Multi-component interventions were common in this review, with the majority of studies adopting this type of approach. Multi-component strategies were more likely to produce successful health outcomes (e.g. increased physical activity or increased vegetable intake) and often paired a motivational strategy with either an environmental, financial or educational strategy. For example, physical activity and healthy eating were increased when motivation was paired with a supportive environment and policy action [40]. Likewise, other researchers agree that multi-component strategies significantly improve staff health outcomes [53, 62]. Most of the multi-component studies incorporated motivational strategies, for example, designated “influential” staff members whose aim was to influence health practices and social norms and tended to increase their own positive health behaviours and considered themselves a role model to other staff [13]. There was no evidence in this review that certain pairings of strategies were more beneficial than other combinations. The heterogeneity of the workforce may require multi-component strategies to attain adequate levels of participant involvement [40].

### Reported barriers in the studies examined

Barriers around time pressures, work schedules, work related fatigue and lack of flexibility in the workplace were frequent [40, 41, 43, 51]. For example, Lemon et al. [40] found that participants on second and third shifts were less likely to take part in intervention activities. The limited capacity of health staff to attend workplace interventions, due to their demanding job roles, may signify that structural components and personalised interventions should be incorporated to increase participation rates [21, 63]. In addition, low participation rates may signal high participant burden [44]. This review observed high intensity activities resulted in decreased participation, and increased participant complaints about heavy workload [40, 43]. Likewise, long interventions, for example maintenance interventions (an intervention following a previous intervention), were suggested to diminish participant motivation to complete the intervention [48]. High participant burden was also observed by a similar systematic review [21]. Nutritional interventions such as food labelling initiatives may not be a burden on participants but can be a burden on workers who deliver the intervention, for example LaCaille et al. referred to how the process of labelling all the foods in the cafeteria was time consuming and some cafeteria employees had low adherence updating the labels [13].

To overcome the barriers listed above, structural measures could be implemented in hospitals to encourage a supportive environment and enhance benefits to staff. Incorporating workplace policy such as decreased healthcare rates or monetary rewards could act as a motivator [40]. The European Network for Workplace Health Promotion recommends that successful dissemination of workplace interventions requires implementation of good policies to ensure organisational commitment is achieved as well as active participation [63]. Furthermore, a universal framework with specified standards for implementation and measuring outcomes is needed to ensure adequate evaluation of programs can be properly conducted [64].

Culture in the workplace can influence participation [65] and management’s flexibility (or inflexibility) regarding intervention strategies can have a significant impact on whether participants are able to actively participate [66, 67]. Having exercise facilities/health club/gym co-located within the hospital setting, may also give staff more flexibility to exercise around shifts. Nutritional interventions such as healthy catering/food policies may also change social and work environment norms to encourage healthy eating. A recent example of this is the Alfred Health, a metropolitan health service in Melbourne, Australia, who implemented state government healthy choice guidelines to improve the healthiness of food options (i.e. retail, vending machines) and catering using the traffic light system and an increase in sales of green/amber (healthier) products and reduction in red (unhealthy) products since implementation [68].

### Limitations of the studies included in this review

The studies in this review were not without their limitations. A number of the studies reviewed presented relatively small sample size in relatively unique hospital environments (e.g. emergency department); and high attrition rates [18, 42, 45]. Concerns of self-selection (e.g. healthy individuals being less interested in health interventions [50] or participants who already have a very high level of physical activity) resulting in a ceiling effect [46] or the healthy worker effect [18, 47]; gender imbalance [50] and the Hawthorne effect need to be considered. The already existing health knowledge of hospital staff may also impact the finding [50].

Managing bias in the sampling process proved to be challenging for many of the studies in this review. Contamination between intervention groups due to close quarters was at times difficult to control [42, 51, 52]. Comparable reviews have stressed that a common downfall of workplace intervention studies is the lack of stringent methodology [27, 69]. Guidelines for an approved methodology and reporting on workplace interventions could be investigated to derive more significant findings in the workplace intervention field.

All the studies in this review included body measurements and the completion of questionnaires in their data collection. Although objective measures are reliable, questionnaires are inherently prone to recall and desirability bias [70]. Participants filling out questionnaires may suffer from inaccurate recall or unconsciously (or consciously) find themselves over-reporting healthy behaviours and under-reporting poor health behaviours. Research shows that individuals often have a difficult time interpreting their own weight status and may feel obliged to record a figure that is closer to social norms [71]. There is a gap in measuring the sustainability of the intervention effects, and future studies should measure for sustained impact in the long term [46] at least 1 year post-intervention.

### Strengths and limitations of this review

A key strength of this review was the systematic approach used to identify studies according to clear inclusion/exclusion criteria. This review’s inclusion criteria aimed to target higher quality evidence by only examining interventions over 12 weeks that used control groups [34] which increases the strength of the findings. Another advantage was that 11/18 studies implemented follow-up periods of over 6 months [13, 18, 39–42, 44, 46, 48, 49, 51]. Sallon et al. [51] state that long interventions increase flexibility by absorbing obstacles and provide a more supportive framework that can account for unanticipated events that arise in hospital environments.

The review has limitations, notably non-English papers were excluded and only six databases were interrogated. Studies published in other languages were excluded and although the most common health databases were searched, the catalogues are not exhaustive of all listings that may publish research on workplace interventions for staff in hospital settings. Hand searching was employed to mitigate this risk and this process identified an additional nine papers. Grey literature was excluded which may limit findings. Two reviewers independently assessed the quality of the studies. Due to the large variance between the studies’ primary outcome measures; comparison of outcomes by meta-analysis was not possible. Similarly, due to the diversity and combination of strategies employed, which parts of the intervention had the greatest effect was not possible.

## Implications and recommendations for future workplace interventions targeting staff in a hospital setting

The various intervention strategies used to improve health outcomes of hospital staff fills a knowledge gap of best practice for workplace interventions. The range in quality of the studies reviewed point to the need for high-quality research in the field. Studies in the current review largely had broad target populations i.e. “hospital employees” and it is recommended to expand future research into specific occupational groups within hospital settings, to fully understand how to best support healthy behaviours in each of these occupational groups (e.g. administration, laboratory, nursing staff etc). Further research would prove beneficial to fully understand the contribution that workplace interventions can have on creating positive behaviour change in staff. As expressed in previous research, agreement on a universal framework or standardised outcome measures could improve applicability of workplace intervention studies and should be investigated further [21, 27]. This addition would also enable meta-analysis to further determine high-quality evidence. The utilisation of structural components, sustainable policy, and context, could also help to achieve high participation rates and improve health outcomes [21, 63].

This review adds to the current body of research as its findings were only derived from studies with a control group and largely consisted of RCTs. Multi-component strategies, motivational strategies, and financial incentives were able to achieve moderate to high quality evidence that workplace interventions significantly improved positive health behaviour change or improved awareness of dietary intake and physical activity habits of staff. These findings concur with other researchers who have found multi-component strategies to be effective at improving health of staff in the workplace [27, 53].

The proportion of health care workers with overweight an obesity is too high. Along with the hospital labour force being largely made up of females; with demanding work tasks and rosters; and declines in physical activity; targeted efforts to promote physical activity [46] as part of a multi-component intervention tackling nutrition and the built environment is needed. The creation of a health promoting workplace is important to achieve a healthy, qualified and motivated workforce; and to achieve this, it will require the participation of employees, management and stakeholders to collectively act on promoting health [6]. An in depth understanding of and enabling staff to overcome the barriers they face in in eating healthy and being physically active is essential to improve the success of workplace interventions in hospital settings, and to improve the health status of hospital staff. Adults spend a large proportion of time in workplace settings. It is therefore critical that workplaces (including physical and psychosocial environments) are conducive to good health [72]. A healthy workplace is achievable with workplace policies supporting good health, supportive environments and evidence-based effective healthy practices [72]. There should be a partnership between employers and employees to protect, promote and sustain good health, safety and wellbeing [72].

## Conclusion

Multi-component strategies, motivational strategies, and financial incentives were particularly effective methods at improving health behaviours of staff in hospital settings. However, difficulty with the heterogeneity of the studies, self-select bias, and some small sample sizes may limit the generalisability of any conclusions. More research is needed from high-quality, randomised control trials to gain further insight into the benefits of workplace interventions aimed at staff in hospital settings. Future studies should focus on incorporating more stringent methodology to address potential bias and longer follow-up time.

## Supplementary Information

**Additional file 1.**

## Data Availability

All data generated or analysed during this study are included in this published article. Full papers from where data was extracted can be found in via Entrez PubMed and other databases listed in the methods section.

## Abbreviations

BMI: Body Mass Index
MVPA: Moderate-to-vigorous physical activity
