# Supplementary material for: Describing workplace interventions aimed to improve health of staff in hospital settings – a systematic review
Source: BMC Health Serv Res. 2022 Apr 7;22:459. doi: 10.1186/s12913-021-07418-9 (PMC8991835; doi:10.1186/s12913-021-07418-9)
Supplement: Supplementary file 1 — Additional file 1. [file 12913_2021_7418_MOESM1_ESM.docx]

Supplementary Table 1: Search strategy

| Search | Search terms |
| --- | --- |
| S1 | nutrition* OR "physical activ*" OR diet* or exercis* |
| S2 | (workplace or worksite) AND (intervention OR "health promotion" OR wellness OR health* OR program*) |
| S3 | staff OR employee* OR provider* OR nurse* OR doctor* OR midwi* OR "shift work" |
| S4 | survey* OR questionnaire* OR “randomi?ed control trial*” OR stud* OR interview* OR “focus group*” |
| S5 | "primary health*" OR hospital* |
| S6 | patient* |
| S7 | S1 AND S2 AND S3 AND S4 AND S5 NOT S6 |
